# Beyond the Maze: Hybrid Ablation and Left Atrial Appendage Occlusion in Cardiac Surgery: Evidence Synthesis and the MESAGE Study Protocol

**DOI:** 10.3390/medicina62050890

**Published:** 2026-05-05

**Authors:** Sotirios C. Kotoulas, Vasileios Kolovos, Nikolaos Tsiamis, Athanasios Kotoulas, Charalampos Georgiou, Panteleimon Tsipas, Ioannis Panagiotou, Dimitrios Antoniadis, Christophoros Kotoulas

**Affiliations:** 1 Department of Cardiothoracic Surgery, 401 General Military Hospital of Athens, Panagiotis Kanellopoulos Avenue, 11525 Athens, Greece; thanassiskot@gmail.com (A.K.); panagiotouioannis@yahoo.gr (I.P.); chrkotoulas@gmail.com (C.K.); 2 Department of Cardiology and Electrophysiology, 401 General Military Hospital of Athens, 11525 Athens, Greece; drkolovos@gmail.com (V.K.); dimitrisantoniadis@hotmail.com (D.A.); 3 Department of Cardiology, Sismanoglio General Hospital, 15126 Athens, Greece; nik.tsiamis@gmail.com

**Keywords:** new technologies, atrial fibrillation, hybrid ablation, surgical ablation, pulmonary vein isolation, posterior wall isolation, BOX lesion, left atrial appendage occlusion, AtriClip, implantable loop recorder

## Abstract

*Background and Objectives*: Atrial fibrillation (AF) is the most common cardiac arrhythmia, present in up to 14–20% of patients undergoing cardiac surgery, with the number of patients expected to double within the next decade. Despite a Class I recommendation for concomitant surgical ablation and a Class I-B recommendation for left atrial appendage (LAA) occlusion in patients with AF undergoing cardiac surgery (Class IIa for endoscopic or hybrid AF ablation), both procedures remain substantially underutilized in clinical practice. The design of the Mapping atrial fibrillation after Epicardial Surgical Ablation plus AtriClip to Guide Endocardial ablation (MESAGE) prospective study is presented. *Materials and Methods*: A narrative literature review was conducted using PubMed through March 2025. Randomized controlled trials, multicenter registries, meta-analyses and current clinical guidelines were prioritized. The MESAGE study protocol is presented in accordance with the SPIRIT recommendations. *Results*: Randomized evidence demonstrates that hybrid ablation achieves 32–48% greater arrhythmia freedom than catheter ablation (CA) alone in persistent and long-standing persistent AF, with comparable safety and significantly fewer interventions at two-year follow-up. Epicardial LAA occlusion with the AtriClip device achieves complete occlusion in all patients with an 87.5% relative reduction in ischemic stroke risk in anticoagulation-free follow-up. Continuous implantable loop recorder (ILR)-based monitoring reveals AF recurrence in substantially more patients than conventional monitoring, with AF burden emerging as a more meaningful endpoint than arrhythmia freedom. The MESAGE study enrolls 40 patients undergoing cardiac surgery who have pre-existing AF, pre-randomized 1:1 to pulmonary vein isolation (PVI) alone versus PVI-BOX, with mandatory pre-operative ILR implantation, intra-operative AtriClip LAA exclusion, and systematic Day-60 endocardial mapping and supplementary ablation using the Affera dual-energy system. *Conclusions*: Hybrid epicardial–endocardial ablation combined with LAA exclusion and continuous ILR monitoring represents a comprehensive, mechanistically rational and evidence-informed approach to AF management in patients undergoing cardiac surgery, although current evidence remains heterogeneous, and the benefits depend on the AF phenotype and monitoring strategy. The MESAGE pilot study will generate hypothesis-generating prospective comparative data on epicardial PVI versus PVI-BOX in the concomitant surgical setting, assessed through systematic post-surgical endocardial mapping and continuous rhythm monitoring.

## 1. Introduction

Atrial fibrillation (AF) is the most common cardiac arrhythmia in clinical practice, with a substantially growing global burden driven by population aging and the rising prevalence of cardiovascular comorbidities [1]. It is associated with a five-fold increase in the risk of ischemic stroke, a three-fold increase in the risk of development of heart failure (HF) and a significant increase in all-cause mortality and hospitalization, imposing major individual and healthcare system costs [1]. Among patients referred for cardiac surgery, AF is present in 14–20%, depending on the procedure type, with the highest rates observed in those undergoing mitral valve surgery or combined coronary and valve procedures.

The 2024 European Society of Cardiology (ESC) Guidelines for the management of AF provide a Class I recommendation for concomitant surgical ablation in patients with AF undergoing cardiac surgery and a Class I recommendation (level of evidence B) for surgical left atrial appendage (LAA) occlusion to reduce thromboembolic risk in patients with AF undergoing cardiac surgery; a separate Class IIa recommendation applies specifically to the endoscopic or hybrid AF ablation setting [1]. Despite this unambiguous guidance, real-world data consistently demonstrate that fewer than 20% of eligible surgical patients receive both interventions simultaneously [2]. This lack of implementation reflects persistent clinical uncertainty on three fundamental pillars: the ablation lesion set that can achieve durable rhythm control; if surgical lesions are reliably complete and transmural; and how post-procedural outcomes should be measured [3].

Conventional post-ablation monitoring with intermittent electrocardiography (ECG) or 24 h Holter recording systemically underestimates AF recurrence by detecting only symptomatic or incidentally documented episodes. The use of an implantable loop recorder (ILR) enables continuous subcutaneous rhythm surveillance, revealing the true AF burden and recurrence patterns that are invisible to standard monitoring, and fundamentally reframes the interpretation of ablation outcomes [4].

Hybrid epicardial–endocardial ablation addresses the substrate limitations of either surgical or catheter-based (CA) ablation alone. Epicardial ablation can create transmural lesions on the posterior left atrial wall, enabling direct management of the LAA and modulating the ganglionic plexi. Endocardial electroanatomical mapping identifies and corrects residual conduction gaps with precision not achievable surgically. The combination of those two modalities provides a mechanistically complementary approach to AF substrate modification, with several randomized trials demonstrating superiority over CA alone in persistent and long-standing persistent AF, although these data coexist with neutral findings from trials using continuous monitoring and have not been uniformly replicated across every outcome measure [5,6,7].

A central unresolved question in the surgical setting remains whether the epicardial pulmonary vein isolation (PVI) alone is sufficient or whether the addition of a posterior wall box-lesion (PVI-BOX) can improve outcomes. This distinction, settled neither by the existing randomized controlled trials data nor by catheter-based trial evidence, carries mechanistic relevance when applied to surgical bipolar clamp devices capable of creating simultaneous transmural box-lesions in a single application.

The MESAGE (Mapping atrial fibrillation after Epicardial Surgical Ablation plus AtriClip to Guide Endocardial ablation) study was designed to address this gap prospectively. It enrolls patients undergoing cardiac surgery with pre-existing AF, pre-randomized 1:1 to epicardial PVI alone versus PVI-BOX, with mandatory pre-operative ILR implantation, intra-operative LAA occlusion with AtriClip (AtriCure, Inc., Mason, OH, USA) and systematic Day-60 endocardial electroanatomical mapping and supplementary ablation using the Affera dual-energy system (Medtronic, Minneapolis, MN, USA). This article presents a narrative review of the evidence underpinning each component of this strategy, followed by the MESAGE study protocol.

## 2. Methods

### 2.1. Literature Review Search Strategy

We conducted a narrative literature review based on targeted searches on PubMed/MEDLINE, Scopus and Web of Science for studies published from January 2010 to June 2025 using keywords including hybrid ablation atrial fibrillation,” “surgical AF ablation cardiac surgery,” “epicardial endocardial ablation,” “pulmonary vein isolation,” “posterior wall isolation,” “left atrial appendage occlusion,” “AtriClip cardiac surgery,” “implantable loop recorder atrial fibrillation,” and “pulsed field ablation.” Reference lists of key articles were also manually reviewed to identify additional relevant publications. Inclusion criteria were studies reporting clinical outcomes, procedural metrics, safety data or rhythm monitoring results related to surgical or hybrid AF ablation, LAA occlusion, or post-ablation monitoring strategies in AF patients. Exclusion criteria were non-human studies and studies not relevant to the cardiac surgery population. The 2024 ESC/EACTS Guidelines for the management of AF are included as key reference documents framing the evidence synthesis [1]. As this is a narrative review, we did not perform quantitative pooling or meta-analysis. No formal risk-of-bias assessment was performed. Study selection and evidence weighing were conducted by author consensus, with priority given to randomized controlled trials, multicenter prospective registries, and meta-analyses.

### 2.2. Study Protocol

The MESAGE study protocol is presented in accordance with the SPIRIT (Standard protocol items: Recommendations for Interventional Trials) guidelines. MESAGE is a single-center, single-blind, prospective, pre-randomized study conducted at the 401 General Military Hospital of Athens, Greece. Ethical approval for the study was granted by the Scientific and Ethics Committee of the 401 General Military Hospital of Athens. Enrollment is ongoing; all patients enrolled have provided written informed consent prior to participation and all future participants will provide informed consent prior to enrollment. No patient data are reported in this manuscript.

## 3. Review of the Literature

### 3.1. Guidelines—The 2024 ESC/EACTS Recommendations

The 2024 ESC guidelines for the management of AF introduced, for the first time, the AF-CARE pathway, which encompasses the following: Comorbidity and risk factor management, patient-centered acute cardiovascular care, Avoid stroke and thromboembolism, Reduce symptoms with rate and rhythm control and Evaluation and dynamic reassessment. It also includes patient empowerment as an integrated, structured framework applicable regardless of AF phenotype or clinical context [1].

Concerning patients undergoing cardiac surgery who are suitable candidates, the recommendation for concomitant surgical ablation in patients with AF is assigned as Class I, level of evidence B. This establishes surgical AF ablation as a routine rather than a selective procedure. Surgical occlusion of the LAA is assigned a Class I recommendation (level of evidence B) in patients with AF undergoing cardiac surgery to reduce thromboembolic risk as an adjunct to oral anticoagulation. This upgrade from the prior Class IIa level, consistent with the 2023 Society of Thoracic Surgeons (STS) Guidelines, which assign a Class I-A recommendation for LAA occlusion in all first-time non-emergent cardiac surgery in AF patients, reflects the growing evidence for epicardial exclusion devices and their potential to complement or replace long-term oral anticoagulation in selected patients. A Class IIa recommendation applies specifically to surgical LAA closure in the endoscopic or hybrid AF ablation setting [1].

Regarding rhythm control strategy, the guidelines favor early intervention with CA or surgical ablation over pharmacological rate control in symptomatic patients. Early rhythm control prevents progressive atrial structural and electrical remodeling and is associated with improved cardiovascular outcomes. PVI remains the cornerstone of AF ablation. However, additional lesion sets, such as posterior wall isolation (PWI), may be considered in patients with more advanced atrial substrate despite the incomplete evidence base for the incremental benefit of such additions [1].

There is a significant implementation gap between real-world practice and guidelines. Multiple datasets all tell the same story: concomitant ablation is performed in 11.9–22.1% of eligible patients, despite a Class I recommendation. Even amongst mitral valvopathy patients, where the evidence is strongest, ablation rates declined by 2.82% per year over an eight-year period [8,9,10,11].

### 3.2. Pathophysiology of AF and the Substrate Basis for Hybrid Ablation

AF is a progressive arrhythmia driven by an interplay between electrical, contractile and structural remodeling of the atrial myocardium. Understanding this progression is essential to appreciating why single-modality ablation strategies are insufficient in advanced disease and why a combined epicardial–endocardial approach addresses the substrate more completely and possibly effectively.

The seminal contribution of Haissaguerre et al. established that spontaneous initiation of AF is predominantly driven by ectopic beats within the pulmonary veins (PVs), which accounted for 94% of identified foci in a landmark series of patients with paroxysmal AF [12]. This was the mechanistic foundation for PVI as the cornerstone of catheter-based AF ablation. In paroxysmal AF, PVI alone achieves more durable rhythm control in the majority of patients, reflecting the dominant role of PV triggers in this phenotype.

As AF progresses from paroxysmal to persistent and ultimately long-standing persistent, the arrhythmogenic substrate evolves substantially. Electrical remodeling, characterized by progressive shortening of the atrial effective refractory period and loss of rate adaptation, develops within the first days of sustained AF and creates conditions that favor re-entry. Contractile remodeling follows, impairing the atrial transport function and significantly contributes to thromboembolic risk by reducing left atrial blood flow velocity and subsequently by structural remodeling, a process lasting weeks to months that includes cardiomyocyte hypertrophy, myolysis, connexin dysregulation and progressive interstitial fibrosis [13]. Both electrical and contractile remodeling are potentially reversible, whereas structural remodeling represents irreversible changes in atrial wall architecture that underlie the stabilization and perpetuation of the fibrillating process.

Kottkamp et al. provided critical insights regarding atrial fibrosis in AF, revealing that it may not be a consequence of the arrhythmia itself but rather represents an independent disease process termed fibrotic atrial cardiomyopathy [14]. Intra-operative biopsy post-mortem, electroanatomical mapping, and delayed enhancement cardiac MRI data consistently demonstrate significant fibrosis even in patients with paroxysmal AF and increasing fibrosis burden in more persistent AF forms. Left atrial volume is closely correlated with fibrosis and spherical remodeling and remains the strongest independent predictor of AF recurrence after CA [15].

An important consideration for the hybrid approach consists of the architecture and composition of the posterior left atrial wall. This region consists of overlapping muscle layers with different fiber orientations, creating acute transitions in conduction that promote block and re-entry even in the absence of significant fibrosis. During the progression of structural remodeling, the posterior wall increasingly functions as an independent substrate region, capable of perpetuating AF without reliance on PV triggers. In this context, structural remodeling is also driven by epicardial adipose tissue, which infiltrates the atrial wall and exerts pro-inflammatory and pro-oxidative effects on the underlying myocardium [16]. This epicardial component is directly accessible during surgical ablation yet entirely inaccessible from the endocardial surface, providing a specific mechanistic rationale for the surgical component of the hybrid strategy. Advanced cardiovascular imaging supports this substrate concept: cardiac computed tomography quantification of peri-atrial epicardial adipose tissue volume, left atrial volume index and coronary fat attenuation index has been independently associated with the presence of AF and may contribute to non-invasive pre-procedural substrate characterization and risk stratification in this population [17].

It is important to highlight the non-PV sources that become dominant contributors to AF maintenance in more persistent AF forms, more specifically, drivers in the coronary sinus, the superior vena cava, the LAA and ganglionic plexi in the base of the PVs and posterior left atrium [3]. These ganglionic plexi, dense networks of autonomic neurons that modulate atrial conduction, are inaccessible endocardially but are directly encountered and ablatable during epicardial surgical procedures. Epicardial access enables transmural posterior left atrial lesion creation, ganglionic plexi modification and simultaneous LAA management, while endocardial electroanatomical mapping identifies and corrects residual conduction gaps with a precision not achievable surgically.

### 3.3. From the Cox-Maze Procedure to Minimally Invasive Hybrid Procedure

The surgical treatment of AF has its origins in the Cox-Maze procedure, first performed in 1987 and described definitively in 1991 [18]. The procedure comprised a series of precisely placed atrial incisions forming an electrical maze that interrupted macro-reentrant circuits while preserving atrial transport function. All seven initial patients were cured of AF without post-operative antiarrhythmic (AAD) treatment, establishing a proof of concept.

Subsequent iterations replaced the cut-and-sew technique with energy-based alternatives, such as radiofrequency (RF) and cryotherapy, which try to replicate the same transmural lesion set with easier accessibility [19]. Cox-Maze IV is the currently accepted iteration, achieving up to 90% freedom from AF at 12 months. Long-term data from patients undergoing the procedure concomitantly with mitral valve surgery confirm > 90% sinus maintenance at one year, declining to 66% off AADs at 7 years with a remarkably low stroke rate of 0.4 events/100 patient-years and 96.6% freedom from embolic stroke at 7 years [20].

Despite this efficacy, adoption rates are low. Technical complexity, the requirement for cardiopulmonary bypass, and risk of sinus node dysfunction limit the adoption [16]. The development of standalone thoracoscopic epicardial ablation on the beating heart addressed these barriers, enabling bilateral PVI, PWI and LAA occlusion through small port incisions without sternotomy or the need for cardiopulmonary bypass. Preclinical data confirmed that a single application of a dedicated RF clamp achieves 100% transmural isolation of the posterior left atrial wall across all sections [21].

The inherent limitation of a purely epicardial approach, however, is the absence of direct electrophysiological insights [16]. The surgeon cannot reliably identify endocardial conduction gaps; certain lesion sets, such as mitral isthmus lines, are inaccessible epicardially; and confirmation of bidirectional block is less reliable than endocardial validation. It was precisely this limitation that motivated the first hybrid concept, first implemented in 2010, by combining the transmural lesion quality of thoracoscopic epicardial surgery with the precision electroanatomical mapping and targeted gap correction of CA.

### 3.4. The Burden of AF in Cardiac Surgery and the Implementation Gap

Patients with AF presenting for cardiac surgery represent a distinct and challenging clinical population. They are older, carry a higher burden of comorbidities, and present with more advanced atrial structural disease than patients without AF undergoing equivalent procedures [2]. AF during cardiac surgery is independently associated with higher operative mortality, increased risk of post-operative stroke, reduced long-term survival and increased rates of HF hospitalization [10]. Therefore, addressing AF at the time of surgery offers the potential to modify the trajectory of the underlying disease.

There is substantial evidence to support concomitant surgical ablation in this population. In a multicenter registry of 807 patients undergoing the Cox-Maze IV procedure concomitantly with cardiac surgery, freedom from AF at 3 years was 87.5% for paroxysmal, 81.9% for persistent and 78.1% for long-standing persistent AF, with 84% of patients off AADs at 3-year follow-up and with no increase in operative mortality compared with expected rates [22]. The German CArdioSurgEry AF registry (CASEF), consisting of 1000 patients, reported sinus rhythm at discharge in 88.1% of standalone and 62.4% of concomitant ablation patients, with a major complication rate of 4.3% in the concomitant group and no deaths in the standalone cohort [23]. Single-center data from 153 patients undergoing concomitant RF ablation with cardiac surgery demonstrated 60% overall freedom from AF at 12 months, with paroxysmal AF and AF history of less than one year identified as independent predictors of procedural success [24]. In patients with persistent AF undergoing non-mitral cardiac surgery, a concomitant Cox-Maze procedure revealed superior five-year survival of 88% versus 64% with PVI alone and significantly lower rate of composite adverse events [10].

Concomitant surgical ablation is, therefore, safe, effective, and prognostically beneficial across AF phenotypes and operative settings. Success depends on lesion set completeness, AF phenotype and arrhythmia duration. More persistent AF forms represent the most challenging substrate.

### 3.5. Hybrid Ablation Studies

Over the past decade, the evidence for hybrid epicardial–endocardial AF ablation has progressed from single-center studies to multicenter randomized controlled trials and formal meta-analytic synthesis, forming an evolving but heterogeneous evidence base. The direction of benefit appears consistent, while the magnitude is influenced by AF phenotype, lesion set, center expertise and the rigor of rhythm monitoring. The summary of evidence for hybrid ablation is summarized in Table 1. The clinical imperative for hybrid ablation is rooted in the well-established limitation of CA alone in this population: single-procedure success rates in persistent and long-standing persistent AF consistently fall below 30% and 50%, even with extensive substrate modification, and the addition of complex fractionated electrogram or linear ablation to PVI does not drastically improve outcomes [3,16].

Randomized controlled trials enrolling patients with persistent and long-standing AF (HARTCAP-AF, CEASE-AF, and CONVERGE) have consistently demonstrated the superiority of hybrid ablation over catheter ablation alone, with absolute freedom-from-arrhythmia benefits ranging from 28 to 48% at 12 months [25]. The CEASE-AF 24-month data confirmed that this advantage is durable [5,6,7,26]. Subanalysis of the CONVERGE LSPAF further demonstrated that patients with the most advanced substrate derived particular benefit, with primary effectiveness of 65.8% vs. 37% at 12 months [25].

The CASA-AF trial is an important methodological benchmark. Using mandatory continuous ILR-based monitoring over 36 months in LSPAF patients, it found no significant difference between surgical and catheter ablation in single-procedure arrhythmia freedom (12% vs. 11%) while presenting comparable AF burden reduction and quality of life between both strategies [27]. The apparent differences between the surgical/hybrid and CA are highly dependent on the rhythm ascertainment method employed. The divergence between CASA-AF [27] on the one hand and CEASE-AF, CONVERGE and recent meta-analytic data on the other reflects genuine heterogeneity in patient selection, lesion set, operator experience and monitoring strategy rather than a settled clinical question.

A meta-analysis from Rivera et al., comprising 358 patients, confirmed a 47% reduction in atrial tachyarrhythmia recurrence with hybrid ablation (RR 0.53, 95% CI, *p* < 0.01) with no significant increase in major adverse events, with trial sequential analysis indicating conclusive evidence [28]. Shrestha et al. reported 69% arrhythmia freedom at one year or later across six Convergent studies with 551 patients, with a 30-day major adverse events rate of 6% [29]. The EHAFA registry provided real-world validation across 468 patients in 17 centers, confirming 79% arrhythmia freedom with AADs and 64% without AADs at one year [30]. Expert consensus best practice guidelines for the hybrid Convergent procedure have further established the importance of a structured surgeon–electrophysiologist team approach, dedicated training programs and systemic procedural optimization as prerequisites for safe and effective program development [31].

**Table 1:** Summary of key hybrid ablation studies in persistent and long-standing persistent atrial fibrillation.

Study	Design	N	Population	Hybrid Approach	Comparator	Follow-Up	Key Results
**HARTCAP-AF** [5]	RCT	41	Persistent/LSPAF	Single stage epicardial PVI, PWI, CTI	CA: PVI, PWI, CTI	12 months	89% vs. 41% freedom from ATA off AAD (*p:* 0.002)
**CEASE-AF** [6,26]	RCT	154	Persistent/LSPAF (LA > 4 cm)	Epicardial PVI, PWI, LAA exclusion	CA: PVI	12 months	71.6% vs. 39.2% freedom from AF/AFL/AT off AAD (*p* < 0.001)
**CEASE-AF 24-month** [27]	RCT follow-up	154	Persistent/LSPAF	As above	CA: PVI	24 months	66.3% vs. 33.3%; reinterventions 18.9% vs. 52.9% (*p* < 0.001)
**CONVERGE LSPAF subanalysis** [25]	RCT post hoc	65	LSPAF	Convergent pericardioscopic epicardial plus endocardial	CA	12 and 18 months	65.8% vs. 37.0% at 12 months (*p* 0.022); 60.5% vs. 25.9% at 18 months (*p:* 0.006)
**CASA-AF** [27]	RCT, ILR monitored	115	LSPAF	Thoracoscopic surgical ablation	CA	36 months	11% vs. 12% single-procedure freedom (HR 1.22; *p:* 0.41); comparable burden reduction and QoL
**Rivera et al. [28]**	Meta-analysis, 3 RCTs	358	Persistent/LSPAF	Hybrid (all types)	CA	12 months	RR 0.53 (95% CI 0.41 to 0.69; *p* < 0.01); no increase in major adverse events
**Shrestha et al.** [29]	Meta-analysis, 6 studies	551	Persistent/LSPAF	Hybrid Convergent	CA	12 or more months	69% arrhythmia freedom (50% off AAD); 30-day MAE 6%
**EHAFA registry** [30]	Prospective registry	468	All AF types (74% non-paroxysmal)	Epicardial and hybrid	N/A	12 months	79% arrhythmia freedom with AAD, 64% without; major complication rate 8.2%
**Makati et al.** [31]	Expert consensus	N/A	Persistent/LSPAF	Hybrid Convergent	N/A	N/A	Best practices for team approach, training, procedural optimisation

AAD, antiarrhythmic drugs; ATA, atrial tachyarrhythmia; CA, catheter ablation; CTI, cavotricuspid isthmus; HR, hazard ratio; LA, left atrium; LAA, left atrial appendage; LSPAF, long-standing persistent atrial fibrillation; MAE, major adverse event; PVI, pulmonary vein isolation; PWI, posterior wall isolation; QoL, quality of life; RCT, randomized controlled trial; RR, risk ratio.

### 3.6. The Lesion Set Debate: PVI-vs-PVI BOX

The question of whether posterior left atrial wall isolation adds incremental benefit is one of the most consequential unresolved questions in AF ablation and has directly influenced the MESAGE study’s design. The posterior wall harbors structurally distinct myocardial architecture prone to re-entry, houses non-PV triggers, and is directly accessible from the epicardial surface during cardiac surgery. The CEASE-AF and HARTCAP-AF hybrid randomized controlled trials incorporated PWI as part of the epicardial lesion set and demonstrated substantial superiority over catheter-based PVI alone [5].

The endocardial evidence is consistently negative. The CAPLA trial with 338 patients with persistent AF found no difference in freedom from arrhythmia at 12 months between PVI + PWI and PVI alone (52.4% versus 53.6%, HR: 0.99, *p*: 0.98), with significantly longer procedural times in the PWI group [32]. This finding was corroborated in the MANIFEST-PF registry of 547 patients undergoing pulse-field ablation (PFA), where adjunctive posterior wall ablation again revealed no benefit in a propensity-matched analysis (71.7% vs. 68.5%. *p*: 0.34) [33]. Even the ADVANTAGE AF Phase 2 trial, which combined PFA PVI and PWI with continuous ILR-based monitoring in 255 patients, found true arrhythmia freedom fell to 52% under continuous monitoring despite 73.4% owing to conventional intermittent assessment [34], reinforcing both the limitations of the endocardial approach and the critical importance of honest endpoint ascertainment.

The consistent failure of endocardial PWI to improve outcomes is mechanistically explicable. Catheter-based lesions, regardless of energy source, struggle to achieve reliable transmurality through the full thickness of the posterior left atrial wall. Epicardial surgical ablation is confirmed to achieve 100% transmurality in preclinical models [21] and is mechanistically better positioned to create durable PWI. An explicit distinction between mechanistic rationale and clinical evidence is therefore warranted. Endocardially, the incremental benefit of PWI has been consistently refuted in randomized controlled trials (CAPLA) and large propensity-matched registries (MANIFEST-PF) and should be interpreted as unsupported by current clinical data. In a surgical context, PWI has been incorporated as part of the epicardial lesion set in the CEASE-AF and HARTCAP-AF within a hybrid strategy rather than an isolated variable [5]. No randomized trial has directly compared epicardial PVI alone against epicardial PVI-BOX within the concomitant surgical setting. The surgical PVI-BOX strategy is backed up by a mechanistic and preclinical rationale, as well as indirect evidence from hybrid trial lesion sets, but not comparative clinical evidence. This the remaining gap is the central question that the MESAGE study is designed to interrogate.

### 3.7. Surgical LAA Occlusion and Stroke Prevention

The LAA is the source of >90% of thrombi responsible for cardioembolic stroke in patients with non-valvular AF. Its occlusion during cardiac surgery addresses a distinct and critical dimension of the AF burden that rhythm control alone cannot fully mitigate, particularly in patients who experience AF recurrence or silent arrhythmia.

The LAAOS III is a landmark trial for concomitant LAA occlusion during cardiac surgery. In total, 4770 patients with AF and a CHA2DS2VASc score of 2 or more who were undergoing cardiac surgery for another indication were randomized, with all participants continuing oral anticoagulation during follow-up. LAA occlusion reduced the risk of ischemic stroke or systemic embolism from 7% to 4.8% over a mean follow-up of 3.8 years. (95% CI 0.53 to 0.85; *p*: 0.001), without any significant increase in perioperative bleeding, HF or mortality [35]. This benefit was observed on top of ongoing anticoagulation in the majority of patients, suggesting that mechanical LAA exclusion provides incremental stroke protection beyond pharmacological therapy alone and may reflect the elimination of a thrombogenic anatomical reservoir that persists despite adequate anticoagulation.

At the device level, epicardial clip-based LAA occlusion achieves complete ostial closure in 100% of cases with durable long-term results confirmed on computed tomography imaging up to 8.1 years post-implant, with no-device complications over a mean follow-up of 26 months in a prospective first-in-man series of 291 patients. In a subgroup of 166 patients who discontinued oral anticoagulation during follow-up, the observed ischemic stroke rate was 0.5/100 patient-years compared with an expected rate of 4/100 patient-years on CHA2DS2-VASc scores, representing an 87.5% relative risk reduction [36]. A prospective series of 144 consecutive patients undergoing thoracoscopic epicardial clip deployment as a standalone or concomitant procedure demonstrated 100% complete LAA occlusion with no mortality or major morbidity and no thromboembolic events over 180 patient-years of anticoagulation-free follow-up [37].

Surgical ligation or stapling techniques carry documented failure rates of 10–40% due to residual LAA stumps or incomplete closure; epicardial clip devices apply circumferential compression at the LAA ostium under direct vision, eliminating residual flow and appendage stump formation. Appropriately, the 2024 ESC/EACTS Guidelines assign a Class I recommendation (level of evidence B) for surgical LAA occlusion in patients with AF undergoing cardiac surgery, while a Class IIa recommendation applies to the endoscopic or hybrid AF ablation setting [1]. In the MESAGE study, all patients will receive an LAA occlusion device, irrespective of the ablation group they are randomized to.

### 3.8. Continuous Rhythm Monitoring with ILR

The accurate assessment of rhythm outcomes after AF ablation depends fundamentally on how monitoring is performed. Conventional post-procedural surveillance relies on ECG at clinical visits, 24 h Holter recordings, or symptom-driven event recorders, leading to systematic underdetection of arrhythmia recurrences from missing all asymptomatic and inter-assessment episodes, inflating apparent procedural success rates and distorting cross-study comparisons. The ILR enables continuous rhythm surveillance for up to 3 years, recording all episodes, regardless of symptoms.

The CIRCA-DOSE trial demonstrated that one-year freedom from atrial tachyarrhythmia owing to continuous ILR-based monitoring amounted to 53% in patients who reported symptomatic freedom rates of approximately 79%, while AF burden was reduced by more than 98% from baseline across all ablation strategies [4]. The ADVANTAGE-AF phase 2 trial found a discrepancy of >20% between conventional intermittent monitoring (73.4%) and continuous ILR-based assessment (52%) in the same persistent AF patients [34].

In the surgical setting, ILR monitoring after concomitant bipolar maze and mitral valve surgery revealed that 4.3% of patients with AF recurrences were completely asymptomatic, and only 27.6% of symptom-triggered patient-reported events were confirmed as genuine AF by concurrent ILR recording [38].

A fundamental problem compounding these monitoring issues is the profound heterogeneity of follow-up protocols across surgical ablation studies. Monitoring modalities, assessment in intervals, blanking period definitions and recurrence thresholds all vary wildly and without standardization, rendering cross-trial comparisons of arrhythmia freedom rates methodologically unreliable and explaining much of the wide variation in reported outcomes across an ostensibly similar literature [4,27,38].

### 3.9. Next-Generation Dual-Energy Mapping and Ablation

The endocardial component of the MESAGE protocol is performed using the Affera mapping and ablation system (Medtronic, Minneapolis, MN, USA), a novel platform that integrates a proprietary three-dimensional electroanatomic mapping system with a focal 9 mm lattice-tip catheter capable of delivering either RF or PFA energy with a single procedure. This dual-energy capability is clinically significant since RF provides precise, temperature-controlled lesion creation with real-time force feedback and established safety characteristics across all anatomical locations, while PF energy applies ultrarapid electrical fields that induce non-thermal, cardiomyocyte-selective tissue injury with a markedly reduced risk of collateral damage to esophagus, phrenic nerve and pulmonary veins [39].

Unipolar endocardial mapping has become a useful adjunct for assessing the durability and transmurality of epicardial lesions after surgical ablation in AF. Direct epicardial assessment post-surgery is often limited, so endocardial mapping is used as a surrogate.

Electroanatomical mapping after epicardial surgical ablation is supported by Bulava et al., who performed systemic electroanatomic mapping on 70 patients with persistent or long-standing persistent AF at a median of 87 days after epicardial thoracoscopic bipolar AF ablation [40]. Only 76% of patients were in sinus rhythm at the time of mapping. Despite intra-operative confirmation of acute conduction block, all pulmonary veins were found to be isolated in only 68.6% of patients at deferred assessment and complete posterior left atrial wall isolation was present in only 22.9%. Left PVs showed significantly higher reconduction rates than right PVs *(75.7% versus 91.4% isolated; p = 0.012).* The roof line required endocardial RF ablation along its entire length in 58.5% of patients, and the most common inferior line gap was located adjacent to the right inferior PV in 62.1% of cases. Acutely confirmed epicardial blocks are frequently transient, and staged deferred endocardial mapping is essential to accurately characterize lesion integrity; the authors conclude that a two-stage hybrid approach is required for effective treatment of persistent AF.

The SmartFIRE study, a prospective multicenter trial, enrolled 149 patients with paroxysmal AF across 14 centers, evaluated the dual-energy focal catheter and demonstrated 100% acute procedural success with first-pass isolation in 89.1% of patients and 96.8% of veins [41]. At mandatory three-month remapping, 87% of treated PV remained durably isolated, with a primary adverse event rate of 4.4% and no esophageal lesions on endoscopy. In a real-world multicenter registry of 130 patients with predominantly non-paroxysmal AF, first-pass isolation of PVs and posterior wall was 100%, with 289 ablation lines performed across diverse anatomical targets and a major complication rate of 1.5%.

In MESAGE, the Day-60 mapping procedure using Affera is primarily a check-up. The electrophysiologist uses the integrated three-dimensional mapping system to characterize the integrity of the epicardial lesion set, identify any residual conduction gaps, and perform targeted supplementary ablation only where gaps are confirmed. This transforms an otherwise empirical CA treatment into a precise, lesion-driven guided intervention directly interrogating the quality of the preceding surgical ablation, following the broader contemporary trend towards tailor-made, patient-specific medicine rather than fixed, empirical treatment strategies.

The evidence supports hybrid epicardial–endocardial AF ablation as a mechanistically rational and clinically attractive strategy for rhythm control in patients with persistent and long-standing persistent AF, with epicardial LAA occlusion providing incremental stroke protection, continuous ILR-based monitoring offering a considerably more reliable assessment of true arrhythmia burden than intermittent methods, and deferred electroanatomical mapping consistently revealing residual conduction gaps after epicardial surgery in the majority of patients. The prospective combined application of this complete multimodal strategy in patients undergoing concomitant cardiac surgery for an independent indication has received limited structured comparative evaluation to date, and the question of whether epicardial PVI alone or PVI with a posterior BOX lesion achieves superior durable rhythm control in this specific population remains unresolved. The MESAGE study was conceived to address this gap.

## 4. The MESAGE Study

**Figure 1:**
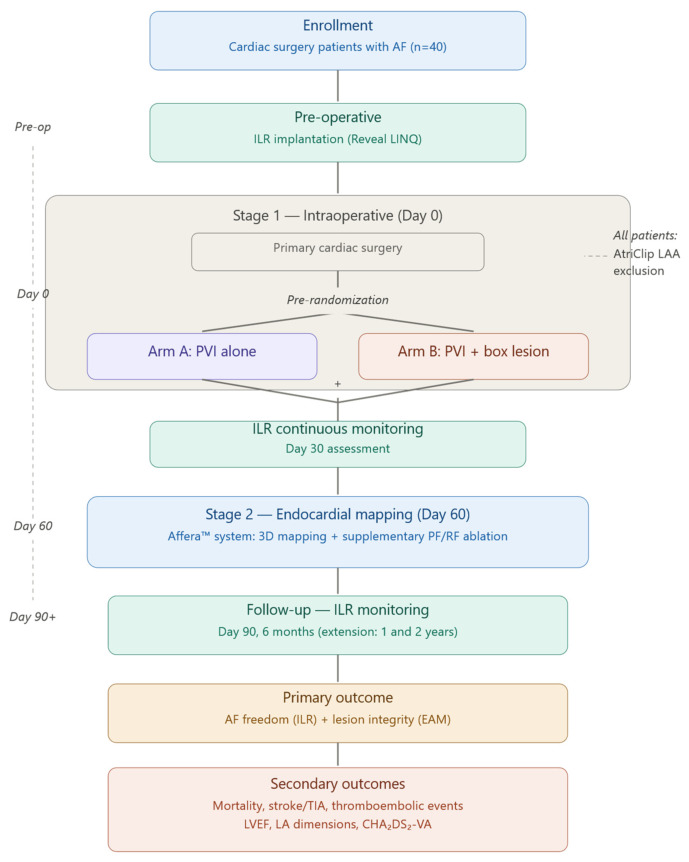
MESAGE protocol study schematic. The diagram summarizes the temporal sequence of study procedures. At the pre-operative stage, all enrolled patients undergo subcutaneous implantable loop recorder (ILR) implantation for baseline AF burden capture and continuous rhythm surveillance throughout the study. Patients are then pre-randomized 1:1 to epicardial pulmonary vein isolation alone (PVI, Arm A) or epicardial PVI with posterior wall BOX lesion (PVI-BOX, Arm B), both performed with bipolar radiofrequency clamps concomitantly with the primary cardiac surgical procedure. All patients receive AtriClip epicardial left atrial appendage exclusion, irrespective of the ablation arm. At Day 60 post-surgery, all patients undergo transcatheter endocardial electroanatomical mapping with the Affera dual-energy system, blinded to lesion set allocation, to verify lesion integrity and perform targeted supplementary ablation of any residual conduction gaps. Rhythm is then followed by continuous ILR monitoring, with outcome assessment at 6, 12 and 24 months.

The MESAGE (Mapping atrial fibrillation after Epicardial Surgical Ablation plus AtriClip to Guide Endocardial ablation) study is a single-center, single-blind, prospective, pre-randomized study conducted at the Department of Cardiothoracic Surgery, 401 General Military Hospital of Athens, Greece. It was designed to address a key knowledge gap regarding whether epicardial PVI alone or PVI-BOX lesion achieves superior durable rhythm control in patients with pre-existing AF undergoing cardiac surgery, assessed through systematic deferred endocardial electroanatomical mapping and continuous ILR-based rhythm monitoring. The study schematic is presented in Figure 1.

### 4.1. Study Population

The study enrolls 40 consecutive patients (all-comers) undergoing cardiac surgery with a documented history of paroxysmal, persistent or long-standing persistent AF. *Inclusion criteria* require subjects to be >18 years of age, with documentation of AF and a planned cardiac surgical procedure amenable to concomitant epicardial ablation. Key baseline variables include CHA2DS2-VA score, age, sex, left ventricular ejection fraction (LVEF), left atrial dimensions, type of primary surgical procedure, AF phenotype and duration and degree of mitral regurgitation. *Exclusion criteria* include prior cardiac surgery involving opening the pericardium or entering the pericardial space, prior LAA occlusion (surgical or percutaneous), planned heart transplant or ventricular assist device implantation, active endocarditis, known allergy to nitinol or nickel sensitivity, known medical condition with life-expectancy of less than year, current enrollment in an interfering investigation, mental impairment precluding informed consent, pregnancy and known severe symptomatic carotid disease. All patients provide informed consent prior to enrollment. AF phenotype (paroxysmal versus persistent/long-standing persistent AF) is recorded at enrollment as a pre-specified baseline characteristic. The rationale for an all-comers, pragmatic enrollment policy is that pre-existing AF of any phenotype constitutes a Class I indication for concomitant surgical ablation under the 2024 ESC/EACTS Guidelines, and a feasibility pilot must reflect the real-world clinical mix encountered at a cardiac surgery unit. To address the well-recognized mechanistic differences between trigger-driven paroxysmal AF and substrate-driven persistent/long-standing persistent AF, outcomes will be reported descriptively within each phenotype as a pre-specified subgroup, with paroxysmal AF treated as a reference subgroup rather than a statistical comparator given the limited anticipated sample in this stratum.

### 4.2. Randomization and Blinding

Patients are pre-randomized 1:1 to one of two epicardial ablation strategies: PVI alone (Arm A) or PVI-BOX (Arm B) using a computer-generated random allocation sequence in permuted blocks, prepared prior to study initiation. Patients are blinded to their ablation assignment. The electrophysiologist performing the Day-60 endocardial procedure is also blinded to the epicardial lesion set, enabling unbiased assessment of lesion integrity and residual conduction gaps. Because the ablation arm determines the intra-operative lesion set, the operating team cannot be blinded. This is an unavoidable feature of any concomitant surgical ablation trial rather than a deliberate design choice. Post-operative care teams and the investigators responsible for ILR interrogation and outcome adjudication are, however, blinded to allocation, and the computerized ILR transmissions are reviewed without knowledge of group assignment to preserve blinded ascertainment of the clinical endpoint.

### 4.3. Interventions

**Pre-operative phase:** All enrolled patients undergo subcutaneous ILR implantation (Reveal LINQ ^TM^ ICM System, Medtronic, Minneapolis, MN, USA) prior to the scheduled cardiac surgery. The ILR serves as the sole monitoring modality for AF recurrence throughout the study, enabling continuous automated rhythm surveillance independent of patient symptoms or scheduled clinical assessments. This pre-operative implantation ensures that baseline AF burden is captured prior to any therapeutic intervention, and was chosen for three specific reasons: it enables each patient to serve as their own within-subject control for AF burden so that post-operative burden can be interpreted against a quantitative pre-intervention baseline rather than a categorical history of AF; it avoids contamination of early post-operative recordings by the known high rate of post-operative supraventricular arrhythmias; and it allows the 90-day blanking period to be defined relative to surgery while still providing uninterrupted automated rhythm surveillance throughout the entire follow-up interval.

**Stage 1—Intra-operative surgical ablation (Day 0**): During surgery, all patients will undergo the primary surgical procedure for their underlying cardiac pathology, followed by concomitant epicardial AF ablation and LAA management. The ablation is performed using bipolar RF clamp technology (AtriCure Inc., Mason, OH, USA). Patients allocated to Arm A receive epicardial PVI using the Isolator Synergy^TM^ Clamps (OLL2/OSL2). Patients allocated to Arm B receive PVI with the same device, followed by posterior wall BOX lesion using the Isolator Synergy EnCompass^TM^ Clamp (AtriCure Inc., Mason, OH, USA). Both procedures are performed on the beating heart without any need for atriotomy, enabling concomitant application during any cardiac surgical procedure that does not require myocardial incision. Irrespective of the ablation arm, all patients receive epicardial LAA exclusion using the AtriClip ^TM^ device (AtriCure Inc., Mason, OH, USA). This standardized component ensures that the thromboembolic risk reduction conferred by mechanical LAA occlusion is uniformly applied across both groups, isolating the effect of the ablation lesion set as the sole between-group variable.

**Stage 2—Endocardial mapping and supplementary ablation (Day 60):** Sixty days post-operation, all patients undergo transcatheter endocardial left-atrial mapping using Affera^TM^ (Medtronic Minneapolis, MN, USA). The 60-day interval was selected to allow for adequate post-surgical recovery, resolution of post-operative inflammation and atrial edema, along with stabilization of ablation lesions before electrophysiological assessment. The Day-60 procedure serves a dual purpose. Firstly, it provides a systematic electrophysiological evaluation of the integrity of the epicardial lesion set, identifying residual conduction gaps across PVI lines and across the posterior wall BOX lesion. Where conduction gaps are identified, the electrophysiologist performs targeted supplementary ablation, thereby converting the empirical surgical lesion set into a verified, complete electrophysiological substrate modification.

### 4.4. Study Outcomes

The *primary outcome* is hierarchical and comprises two complementary but conceptually distinct domains. The primary clinical endpoint is ILR-confirmed freedom from any atrial tachyarrhythmia (AF, atrial flutter or atrial tachycardia) of ≥30 s duration, off class I or III antiarrhythmic drugs, after a 90-day post-surgical blanking period, evaluated at 12 months. The co-primary mechanistic endpoint is the integrity of the epicardial lesion set at Day-60 transcatheter endocardial electroanatomical mapping, defined as persistent bidirectional conduction block across each targeted line (pulmonary veins and, in Arm B, the posterior wall-BOX). AF burden, expressed as a percentage of monitored time in AF on continuous ILR, will be analyzed as a quantitative secondary endpoint and as a prespecified alternative rhythm-control metric, given its documented superiority to binary freedom from arrhythmia in contemporary AF trials. The two primary domains are reported separately and not pooled into a single composite score.

*Secondary outcomes* include all-cause mortality at one year, occurrence of transient ischemic attack (TIA) or stroke, occurrence of thromboembolic events, improvement in cardiac markers, including LVEF, left atrial dimensions, any additional unanticipated surgical or interventional procedure related to the device or the procedure performed to prevent a life-threatening or permanently disabling event and CHA2DS2-VA score evaluation. Additional peri-procedural data collected include intensive care unit (ICU) length of stay, units of blood transfusion and incidence of renal failure. Long-term outcomes evaluated at the six-month, one-year and two-year intervals include lesion-related mortality, LVEF change, disabling stroke, and any additional unanticipated surgical or interventional procedure performed to prevent a life-threatening or permanently disabling event.

### 4.5. Statistical Considerations

MESAGE is a proof-of-concept pilot study. The sample size of 40 patients is consistent with established recommendations for pilot studies, which suggest that 15 to 20 participants per group is sufficient to estimate variability, assess feasibility and inform the design of a subsequent definitive trial [42]. Descriptive statistics will be used to characterize baseline demographics, procedural variables and outcome data. Continuous variables will be presented as mean ± standard deviation or median with interquartile range as appropriate. Categorical variables will be expressed as frequencies and percentages. Between-group comparisons will be performed using the χ-squared or Fisher’s exact test for categorical variables. Freedom from AF will be analyzed using the Kaplan–Meier survival estimates, and between-group differences will be assessed with the log-rank test.

MESAGE is explicitly designed as a feasibility and hypothesis-generating pilot. No formal power calculation for superiority between arms has been performed, and none is appropriate at this stage because the objective is to estimate the variability of clinical and mechanistic endpoints, quantify the rate and anatomical distribution of residual conduction gaps after epicardial PVI alone versus PVI-BOX, characterize ILR-derived AF burden trajectories in this population and generate the effect-size estimates needed to inform the sample size of a subsequent adequately powered multicenter randomized trial. Between-arm comparisons reported from MESAGE should therefore be interpreted as exploratory and as inputs to trial design, not as definitive tests of clinical superiority.

### 4.6. External Validity and Reproducibility

Several structural features of the MESAGE design must be acknowledged when considering its external validity. The study is single-center, is conducted by a dedicated joint cardiothoracic surgery–electrophysiology team, and relies on specific device platforms. These choices reflect the current standard of care at our institution and the technologies with the most mature supporting evidence at the time of protocol design, but they also constitute a reproducibility constraint. Findings from MESAGE should therefore be regarded as applicable, in the first instance, to tertiary cardiac surgery units with established hybrid AF programs and access to equivalent device ecosystems, and not automatically generalizable to centers operating with different technologies, lower hybrid case volumes or without an integrated electrophysiology service. If the initial signals support a definitive trial, a multicenter design incorporating heterogeneous center experience and alternative device platforms will be required to establish the generalizability of the strategy across the broader cardiac surgery community.

## 5. Conclusions

Hybrid epicardial–endocardial ablation has been associated with substantially greater arrhythmia freedom than CA alone in persistent and long-standing persistent AF in several randomized trials; epicardial LAA occlusion with AtriClip achieves reliable anatomical closure with meaningful stroke risk reduction, and continuous ILR monitoring reveals the true AF burden that conventional follow-up systematically underestimates. These components are all endorsed by the 2024 ESC/EACTS Guidelines yet remain markedly underutilized in real-world cardiac surgery practice. At the same time, the evidence base is heterogeneous: the magnitude of benefit varies with AF phenotype, lesion set, monitoring strategy and center expertise, and at least one randomized trial with mandatory continuous monitoring (CASA-AF) has not shown superiority of the surgical over the catheter-based strategy in long-standing persistent AF [27]. The conclusions drawn in this review should be interpreted considering this uncertainty.

The prospective application of a complete multimodal strategy comprising epicardial ablation, LAA occlusion and continuous ILR monitoring in patients undergoing cardiac surgery with pre-existing AF has received limited structured comparative evaluation to date. The MESAGE pilot study will generate, to the best of our knowledge, among the first prospective comparative data on epicardial PVI alone versus PVI-BOX in the concomitant surgical setting, with lesion integrity verified through systematic Day-60 endocardial electroanatomical mapping and rhythm outcomes captured by continuous ILR surveillance. Given its single-center, technology-specific and feasibility design, MESAGE is intended to inform lesion set selection and to support the rational design of a subsequent adequately powered multicenter trial rather than to deliver a definitive clinical answer on its own.

## Data Availability

This manuscript presents a narrative review and study protocol; no new patient-level data are reported. All primary and secondary literature informing the review is publicly available on PubMed, and data generated by the MESAGE study, once available, will be provided by the corresponding author upon reasonable request, subject to institutional and ethics committee approval.

## Abbreviations

The following abbreviations are used in this manuscript:

AAD	Antiarrhythmic Drug
AF	Atrial Fibrillation
AFL	Atrial Flutter
AT	Atrial Tachycardia
ATA	Atrial Tachyarrhythmia
CA	Catheter Ablation
CI	Confidence Interval
CTI	Cavo-tricuspid Isthmus
ECG	Electrocardiography
HF	Heart Failure
HR	Hazard Ratio
ICU	Intensive Care Unit
ILR	Implantable Loop Recorder
LA	Left Atrium
LAA	Left Atrial Appendage
LSPAF	Long-Standing Persistent Atrial Fibrillation
MAE	Major Adverse Event
MRI	Magnetic Resonance Imaging
PF/PFA	Pulse Field Ablation
PV	Pulmonary Vein
PVI	Pulmonary Vein Isolation
PVI-BOX	Pulmonary Vein Isolation Plus Posterior Wall Box Lesion
PWI	Posterior Wall Isolation
QoL	Quality of Life
RCT	Randomized Controlled Trial
RF	Radiofrequency
RR	Risk Ratio
SPIRIT	Standard Protocol Items: Recommendations for Interventional Trials
STS	Society of Thoracic Surgeons
TIA	Transient Ischemic Attack
